# Supplementary material for: Are carbon emissions trading and green financial instruments synergistic? -Comprehensive quantitative research based on content analysis
Source: PLoS One. 2024 Mar 7;19(3):e0298601. doi: 10.1371/journal.pone.0298601 (PMC10919601; doi:10.1371/journal.pone.0298601)
Supplement: S1 Data — (ZIP) [file pone.0298601.s001.zip › Data/地级市Z_F工作报告.zip!/╜¡╦╒/╨∞╓▌╩╨/└┤╫╘╣1⁄2╓┌║┼í╛┬φ┐╦╩2╛▌═°í┐_2016.docx]

各位代表：

现在，我代表市人民政府向大会作工作报告，请予审议，并请市政协各位委员和其他列席人员提出意见。

一、“十二五”及2015年经济社会发展回顾

“十二五”时期，是宏观形势错综复杂、各种矛盾困难叠加、经济下行压力加大、改革发展任务艰巨繁重的五年，也是徐州各项发展取得重大成就、老工业基地振兴取得显著成效、全面小康社会建设取得阶段性成果的五年。五年来，在省委省政府和市委的正确领导下，全市上下认真贯彻落实党的十八大和十八届三中、四中、五中全会精神，凝心聚力，开拓奋进，深入实施“八项工程”，全力推进“三重一大”建设，顺利完成“十二五”规划确定的主要目标任务。

（一）致力于推动转型升级，经济结构在扩量提质中明显优化。

综合实力迈上更高台阶。“十二五”期间，全市经济保持平稳较快增长，主要指标增速继续高于全国全省。2015年地区生产总值预计（下同）实现5320亿元，五年年均增长11.7%；一般公共预算收入完成530.7亿元，是2010年的2.4倍；固定资产投资达到4277亿元，总量实现翻一番；社会消费品零售总额实现2357亿元，为“十一五”末的2.5倍。县域经济明显增强，主要指标增速连续九年高于全市平均水平，“丰县崛起”和“突破睢宁”战略扎实推进，铜山、邳州、沛县、新沂在全国百强县排名持续提升。民营经济加快发展，全市私营企业达到12.1万户，五年新增7.6万户。

产业发展层次显著提升。三次产业结构由2010年的9.6∶50.7∶39.7演变为2015年的9.5∶44.5∶46，规模以上工业增加值实现翻番，服务业比重累计提高6.3个百分点，高新技术产业占比由20.8%提高到35.5%。省级高新技术企业由93家增至253家，限额以上商贸流通企业由1151家增至2648家。新增上市企业8家，总数达11家；中小企业场外市场挂牌118家，居全省第4位。园区承载能力进一步增强，徐州经济技术开发区成为江北首家国家生态工业示范园区，高新区晋级苏北首家国家高新区。农业生产继续保持良好态势，粮食总产实现“十二连丰”，新增高效设施农业78.8万亩，总面积和占比稳居全省第一。

项目建设取得重大进展。五年来，累计实施亿元以上项目1308个，其中重大项目585个、投入资金4100亿元。亿吨大港一期、湖西航道一期等一批重大基础设施项目竣工使用，郑徐客专、骆马湖第二水源地等项目开工建设，黄河故道综合开发、空军机场迁建等项目进展顺利。徐工重型汽车、考伯斯煤焦油一体化、必康新医药等一批重大制造业项目建成投产，苏宁商务广场等服务业重大项目实现主体竣工。

（二）致力于深化改革开放，发展活力在创新创优中显著增强。

各项改革深入推进。编制完成政府行政权力、行政审批事项、政府专项资金管理、行政性事业收费、投资审批负面清单“五张清单”及政府责任清单目录，市级行政审批项目精简幅度达39%，非行政审批项目全面取消。新一轮市县政府机构改革顺利完成，国家级综合行政执法体制改革试点、省级经济发达镇行政管理体制改革试点、市级扩权强镇试点及街道办事处管理体制改革有序实施。实行企业设立“三证合一”、“一照一码”，企业投资项目审批时限由110天缩短到65天左右。国有企业改革扎实推进。党政机关公车改革基本完成。农村土地承包经营权确权登记颁证工作稳步开展。全国不动产登记试点顺利进行，颁发全国第一本不动产权证书。综合医改试点全面推开。

对外开放深度拓展。五年来，累计完成实际到帐注册外资77.8亿美元、进出口321.7亿美元，分别是“十一五”的2.6倍和2.3倍。企业“走出去”步伐加快，徐工巴西制造基地等一批项目成功落地。保税物流中心通过省级预验收。“徐新亚”国际货运班列顺利开行。观音机场晋升国家级一类航空口岸，新开通国际航线3条、港澳台航线3条。徐州成为苏北首个市民赴台个人游城市。

重点领域创新成效突出。累计申请专利7.9万件、授权4.2万件，是“十一五”的近4倍。新增专业技术人才10.9万人、高技能人才10.8万人。建成大学生创业园20个，大学生创业企业达到6000余家。直接融资占比提高到30%以上，设立多支股权基金，16个项目列入国家和省PPP融资示范项目。黄河故道流域土地综合整治列入省节约集约用地试点。我市被列为全国智慧城市建设试点市、电子商务示范市、信息消费试点市。“智慧徐州”、信用体系建设全面展开。

（三）致力于城乡统筹发展，“1530”城镇体系在协调推进中稳步构建。

中心城市功能不断提升。完成城市总体规划修编。累计实施城建重点工程809项，实现投资1700多亿元。三环东路高架、西路高架等道路建成通车，城市轨道交通、三环北路高架等重大项目加快实施，奥体中心、云龙万达广场、刘湾水厂扩建等一批功能性项目竣工使用，各项设施配套更加完善。珠山景区、潘安湖湿地等精品园林相继建成并向市民开放，5000平方米以上公园绿地达到177个，基本建成公园绿地市民出行“10分钟服务圈”。机电动三（四）轮车以及占道经营、户外广告、老旧小区综合整治成效明显，建成省级城市管理示范社区5个、示范路17条、“幸福家园”36个。新增国家A级景区50家，云龙湖5A级景区创建通过景观价值评审，建成徐州旅游服务中心。先后荣获国家环保模范城市、国家森林城市、国家卫生城市、国家生态园林城市等称号，赢得了“一城青山半城湖”的美誉。

县域城镇化进程明显加快。5个县（市）新城建设和老城改造同步推进，建成区面积实现翻番。30个特色鲜明的中心镇基本形成，经济总量和财政收入分别达到创建初的2.5倍和3.5倍，镇区规模扩张和人口集聚明显加快。管理示范镇创建活动深入实施，完成30个镇验收命名。全市城镇化率由53.9%提高到61%，其中县域城镇化率由44.2%提高到51.1%。

新农村建设进展顺利。新增高标准农田110万亩，有效灌溉面积达768万亩。250.5万农村人口饮水安全问题得到解决。新建改建农村公路2361公里、农村小公益桥梁3244座，2178个行政村全部通达客运班车。改造农村危房3.3万户。新建改建各类无害化厕所55.8万座。完成10375个村庄环境整治。创建省三星级“康居乡村”70个，省“美丽乡村”示范村46个。

（四）致力于改善生态环境，美丽徐州新形象在绿色发展中日益彰显。

生态修复有力有效。“五大行动计划”深入实施。“二次进军荒山”达到预期效果，累计新增造林65.5万亩，林木覆盖率提高到32.5%。市区建成区绿化覆盖率达43.6%，由全省第7位跃居第2位。通过农村居民点整理、增减挂钩新增耕地11.8万亩，复垦工矿废弃地和采煤塌陷地18万亩，盘活存量土地8.3万亩。我市被列入全国首批水生态文明建设试点市，获批国家级水利风景区6个、省级17个。生态红线区域保护规划编制实施。省级生态市创建通过验收。

污染防治持续强化。累计完成大气污染治理工程267项，市区PM2.5浓度较2013年累计下降15.6%。依法关闭取缔小炼钢企业57家，主城区3家热电企业实施撤并整合，三环路以内58家工业企业实现退城入园，秸秆禁烧和综合利用全面加强。市区新扩建污水处理厂7座，新增镇级污水处理厂91座。水环境功能区Ⅲ类以上水体占比达79%，五年提高33个百分点，重点断面水质稳定达标。城乡生活垃圾收运处置体系建成运行，镇村生活垃圾集中收运率提升到95%。

节能减排成效显著。实施节能技术改造项目422个，314家年耗能3000吨标煤企业、396家年耗电1000万千瓦时以上企业完成节能改造。单位GDP能耗累计下降23.5%左右，化学需氧量、氨氮、二氧化硫、氮氧化物排放削减完成省定任务。污染物排放指标有偿分配和排污权交易机制初步建立。获批创建国家节能减排财政政策综合示范市。

（五）致力于改善民生民计，群众生活水平在共建共享中加速提升。

民生实事项目全面落实。五年累计实施棚户区改造、街坊中心建设等实事工程416件。城乡居民收入分别达26150元和14100元，年均分别增长11%和13.2%。实现87.7万年收入4000元以下人口脱贫和359个经济薄弱村新“八有”目标。退休职工人均养老金达每月2245元，较“十一五”末增长67.2%。新增养老床位2.8万张，每千名老年人拥有养老床位32.7张。尊老金发放惠及22.7万高龄老年人。实行60岁以上老年人半价、70岁以上老年人免费乘坐市内公交车。累计实施市区棚户区改造2750万平方米，打造出棚改“徐州模式”。建设保障房近4万套，办理住房公积金贷款和提取353亿元。建成智能公交系统，购置和更新公交车2191辆。

就业服务和社会保障体系日益完善。五年累计新增城镇就业56.9万人、失业人员再就业36.8万人、农村劳动力转移30.9万人，城镇登记失业率控制在1.9%。社会保障主要险种参保率均超过95%，建立了职工医保、居民医保“二次补助”长效机制，新农合跨省异地就医联网结报工作经验面向全国推广。城乡低保惠及18.35万困难群众，临时生活救助、医疗救助以及残疾人系列救助活动扎实开展。

各项社会事业协调发展。新改扩建中小学223所、幼儿园502所，加固、重建校舍454万平方米。所有县（市）区全部通过“全国义务教育基本均衡县市区”验收。幼师、生物工程学院成功升专，徐州工程学院完成新校区建设。医院布局调整加快实施，新增三级医院10家、总数达到16家，完成新一轮社区卫生服务机构提档升级，基本药物制度实现全覆盖。龟山汉墓、博物馆等一批文化设施完成升级改造，“舞动汉风”工程、“书香徐州”建设、文艺精品创作、“好人文化”建设成果丰硕。成功举办第18届省运会，全民健身蓬勃开展，跻身省体育强市，获评全国地市级武术之乡。获批全国文明城市提名城市。双拥、人防、国防动员、民族宗教、档案、外事、侨务、台湾事务、物价、新闻出版、无线电管理、淮塔管理、防震减灾、气象、青少年、妇女儿童、关心下一代等工作都取得了新进步。

社会大局保持和谐稳定。社会治理创新工程深入实施，平安徐州、法治徐州建设取得显著成效。大调解专业化规范化建设、涉法涉诉信访改革、社会面巡防体制重组、行政执法与刑事司法衔接、社会矛盾纠纷排查化解、12348公共法律服务平台建设等走在全省前列，刑事案件发案逐年下降。安全生产责任体系、应急体系、食品药品监管体系进一步完善，安全生产事故起数和死亡人数连续5年双下降。

（六）致力于提升服务效能，政府自身建设在职能转变中全面加强。

政务服务水平明显提升。审批流程完成优化再造，审批环节优化幅度、时限压缩幅度分别达到44%和52.8%，网上审批平台、投资项目在线审批平台建设加快推进。集审批服务、公共服务、便民服务和公共资源交易“四位一体”的政务服务体系初步形成，市政务服务中心荣获全国行政服务大厅“百优十佳”称号。政务协同办公系统实现政府部门全覆盖，网络化政务办公机制逐步建立。百姓办事“零障碍”工程深入实施，累计清理办事障碍5290项，优化办事流程1580项。12345政府服务热线累计接处群众各类诉求109.2万件，办结率98.6%。

行政权力运行更加规范。法治政府建设不断强化，领导干部集体学法实现制度化，行政决策、行政执法规范化建设有序推进，集新闻发布、政务微博等功能于一体的“徐州发布”平台上线运行。市直部门预决算全部公开，2015年“三公”经费比2010年下降30%以上。自觉接受市人大及其常委会的法律监督和工作监督、市政协及社会各界的民主监督，累计办理人大代表建议1282件、政协提案2187件，答复率100%，满意率97%以上。

廉洁政府建设不断加强。严格落实中央“八项规定”，深入开展专项整治，撤销各类领导小组和协调机构1154个，清退办公用房14.3万平方米，处理“吃空饷”1451人。扎实推进政府系统党的群众路线教育实践活动和“三严三实”专题教育。不断加强行风政风建设和审计监督，严查“四风”和腐败问题，对不作为、乱作为的322人进行了问责处理，一批贪污腐败分子受到惩处。

“十二五”期间的发展成绩，是全市人民团结拼搏的结果，也是社会各界共同努力的结果。在此，我代表市人民政府，向全市各族人民群众，向人民解放军驻徐部队、武警官兵和公安民警，向各民主党派、工商联、无党派人士和人民团体，向支持徐州建设的香港特别行政区同胞、澳门特别行政区同胞、台湾同胞、海外侨胞和各界人士，向投身徐州经济社会发展的国内外投资者和企业家，表示衷心的感谢和崇高的敬意！

在肯定成绩的同时，我们也清醒地看到，虽然我市近几年的发展取得了很大成绩，但发展不充分、不平衡、不协调、不可持续的问题依然突出；经济增速相对放缓，产业竞争力不强、创新能力不足、城乡二元结构明显、资源约束趋紧、生态环境脆弱的现状没有根本改变，转方式调结构的任务依然艰巨；居民稳定增收难度加大，基本公共服务供给不足，人口老龄化加快，改善民生任重道远；少数政府部门和工作人员服务意识不强，转变作风、反腐倡廉建设仍需进一步加强。我们一定高度重视这些问题，采取有力有效措施，切实加以解决。

二、“十三五”时期的奋斗目标和战略部署

“十三五”时期，是徐州深化改革开放、加快经济发展方式转变的攻坚阶段，也是我市加快推动老工业基地振兴、在苏北率先全面建成小康社会的关键阶段。

抓好今后五年的工作，必须正确认识经济新常态，把握好发展的“大逻辑”。主动适应经济从高速转向中高速增长，发展方式从规模速度型粗放增长转向质量效率型集约增长，经济结构从中低端转向中高端，发展动力从要素驱动、投资驱动转向创新驱动的新特征，集中精力加快转变发展方式、调整经济结构、培育新的增长点，努力抓住新常态下的战略机遇期，在开拓奋进中实现“弯道超越”。必须认真贯彻五大发展理念，提升增长的质量和效益。将创新、协调、绿色、开放、共享五大理念贯穿于发展全过程，认真落实供给侧结构性改革要求，更加注重解决好发展动力接续、发展不够平衡、人与自然和谐、发展内外联动、民生共建共享等问题，切实以新理念引领发展、破解难题、厚植优势，努力实现更高质量、更有效率、更加公平、更可持续的增长。必须牢固树立统筹协调的观念，增强发展的整体性和系统性。树立正确的价值观和方法论，全面考虑徐州的发展阶段、发展目标、发展重点，统筹经济发展和生态建设、民生改善的关系，稳增长和调结构的关系，创新驱动和投资拉动的关系，传统产业和新兴产业的关系，制造业和服务业的关系，工业化和城镇化的关系，城镇建设和农村发展的关系，实现经济社会的全面发展和全面进步。必须大力推进改革开放创新，加快培育发展新动能。打好全面深化改革攻坚战，拓展对内对外开放空间，推进理念创新、机制创新、方法创新和科技人才创新，更大范围、更高层次集聚发展要素，使改革开放创新成为推动发展的强劲引擎。必须始终坚持以人为本，不断增强人民群众获得感。让徐州人民过上更加幸福的生活，是政府工作的出发点和落脚点。要顺应群众过上美好生活的新期待，围绕“七个更”的要求，集中更多财力、精力用于改善和保障民生，使徐州转型发展、提升发展的过程成为全市人民群众得实惠的过程，巩固形成发展人人共创、成果人人共享、和谐人人共建的良好局面。

今后五年，我市经济社会发展的主要目标是，在苏北率先建成全面小康社会，打造经济强、百姓富、环境美、社会文明程度高的新徐州。

——建设综合实力明显增强、产业发展水平更高的新徐州。地区生产总值年均增长8.5%左右，到2020年人均GDP达到9.5万元左右。服务业占比达到50%左右，科技研发投入占比达到2.5%左右，农业现代化水平明显提高，现代产业体系基本形成。

——建设生活质量显著提升、人民群众更幸福的新徐州。到2020年人均收入较2010年翻一番，完成新一轮脱贫攻坚。就业更加充分，社会保障全民覆盖，基本公共服务体系更加健全，文化产业和文化事业繁荣发展。公民思想道德素质、科学文化素质和公众安全感明显提升。

——建设城市功能全面提高、城乡发展更协调的新徐州。全市城镇化率提高到68%左右，中心城市辐射带动作用和县域经济实力持续增强，加快建设5个中等城市和一批各具特色的中小城镇群，农村生产生活条件明显改善，统筹发展水平、城乡一体化水平显著提升。

——建设生态面貌大幅改善、人居环境更美好的新徐州。空气质量二级标准以上天数比例达到70%，污染治理、节能减排和生态保护修复取得明显成效，城乡环境面貌不断提升，实现天更蓝、地更绿、水更清、路更畅、城更靓。

——建设发展活力竞相迸发、体制机制更完善的新徐州。在重要领域和关键环节改革创新上取得重要成果，形成全方位对内对外开放的新格局。法治政府建设深入推进、公信力明显提高，诚信体系建设实现突破性进展，形成便利的投资营商环境、良好的创新创业环境。

围绕实现上述奋斗目标，必须进一步增强加快发展的责任感和紧迫感，积极适应新常态、引领新常态，高点定位，自我加压，埋头实干，主动作为，带动全市上下一体发力、共同奋斗，开创徐州科学发展、转型发展、提升发展的新局面！

三、2016年政府工作的主要目标任务

今年是全面建成小康社会决胜阶段的开局之年，也是推进结构性改革的攻坚之年。全市经济社会发展的主要预期目标是：地区生产总值增长9%左右，一般公共预算收入增长9%左右，固定资产投资增长15%左右，社会消费品零售总额增长11%左右，实际到帐注册外资和进出口总额保持增长，居民人均可支配收入与经济同步增长，科技研发投入占地区生产总值比重达2.1%左右，居民消费价格涨幅控制在3%左右，城镇登记失业率控制在4%以内，人口自然增长率控制在8‰以内，节能减排确保完成省定任务。

新的一年里，要密切关注宏观经济形势变化，顺应国家政策导向，牢牢抓住发展第一要务，坚持稳中求进工作总基调，把稳增长作为基本目标，认真落实“十个更加注重”的要求，突出供给侧结构性改革，落实好去产能、去库存、去杠杆、降成本、补短板五大任务，推动经济持续平稳健康发展，实现“十三五”良好开局。重点抓好以下七个方面的工作：

（一）以构建现代产业体系为目标，不断调整优化产业结构，做大做强实体经济。产业是发展之本、兴市之基、就业之源，产业发展是提升徐州区域竞争力的核心和关键。要坚持传统产业和新兴产业并重，优化存量、引导增量并举，加快构建以先进制造业为主体、战略性新兴产业为先导、现代服务业为支撑、现代农业为基础的现代产业体系。

更大力度壮大提升主导产业。传统产业是主力军、主税源，新兴产业是增长点、爆发点。要加快改造升级传统产业，以高端化、集群化、绿色化为导向，实施质量品牌提升行动，着力推进技术升级、设备更新、低碳改造，加强战略合作、兼并重组，促进工程机械、煤盐化工、冶金、建材业迈向产业链高端。加速培育新兴产业，制定实施战略性新兴产业培育发展规划和推进方案，优先发展新能源、高端装备两大优势产业，提升发展新能源汽车、光伏、新材料、电子商务、生物医药产业，培育发展大数据、物联网、节能环保产业，壮大金融服务、现代物流、商务服务、检验检测等现代服务业。制定实施“中国制造2025”徐州行动纲要，以延长补齐加粗产业链条为路径，抓好关键技术攻关、自主品牌创建、核心产品推广和两化深度融合，构建区域性现代制造业高地。切实抓好去产能工作，积极运用市场机制、经济手段和法治办法，分业分类化解过剩产能、淘汰落后产能。

加快推进现代农业提档升级。农业丰则基础强，农民富则徐州富。要以保障农产品安全有效供给和促进农民增收为核心，加快农业现代化进程，促进农业转方式、调结构、强基础。继续稳定粮食生产、调整种养结构，提高粮食品质和农业机械化水平。加快发展高效设施农业，年内新增设施农业面积10万亩、改造升级10万亩。培育新型农业经营主体，支持专业种养大户、家庭农场、农民合作社发展。健全完善土地流转机制，推进农村土地承包经营权流转，推广股田制、农田托管经营等新模式，推动农业生产由分散经营向适度规模经营转变。开展“互联网+现代农业”行动，支持农业龙头企业发展壮大，提升农业标准化、产业化水平，促进农村一二三产业融合发展。深入实施黄河故道“七位一体”综合开发，打造国家级现代农业示范区。积极做好农业普查。

充分发挥企业的发展主体作用。企业是发展的主体。企业强，徐州则强；企业发展，徐州才能发展。要大力优化企业服务，健全包挂联系重点企业制度，深化政银企对接活动，以优质高效的政府服务为企业营造良好的发展“小气候”。坚持政企联手、共克时艰，及时掌握重点行业的运行态势、重点企业的经营状况、重点产品的市场变化，加大资金、土地、煤电气、劳动力等要素协调力度，加强企业债务风险防控，帮助企业开拓市场、稳定生产，用实际行动为企业排忧解难。落实国家减税清费政策，切实降低制度性交易成本、人工成本、企业税费负担、社会保险费、企业财务成本、电力价格、物流成本，为困难企业“松绑助力”、为优质企业“加油鼓劲”。建立健全激励机制，提高政策“精准度”和“含金量”，引导企业加强科技创新、商业模式创新、融资方式创新和治理能力创新，加快转变生产经营方式，强化科技投入和人才引进，鼓励优势企业兼并重组其他关联企业。

集中精力主攻项目建设。项目是发展的“牛鼻子”。要始终把项目投入作为稳增长强经济的支撑点、作为调结构促转型的主抓手，进一步增强项目意识、突出项目导向，抓好项目引进、储备、审批，健全项目推进和要素保障机制，在全市上下形成全力以赴抓项目、促发展的浓厚氛围。计划实施重大项目180项、总投资3611亿元，其中年度实施156项、投资739亿元。重点推进徐工大型桩工机械、淮海控股新能源汽车、圣戈班搬迁、徐烟异地技改、正大食品、晶玻光学触控屏等制造业项目，苏宁商务广场、铜山万达广场、万科淮海新天地、雨润农副产品全球采购中心等重大服务业项目，力争早建成、早投产。全力抓好协鑫中能半导体级多晶硅、苏氏精密铸造等前期推进项目，力争早落地、早开工。

（二）以打造淮海经济区中心城市为目标，不断完善城市功能，提高城市的集聚力辐射力带动力。认真落实中央城市工作会议精神，充分发挥城市的区位优势、规模优势和基础优势，坚持产业结构和空间结构联动优化、功能布局与人口分布相互协调、服务设施和管理机制同步完善，全面提升城市品质、能级、市容市貌。

优化城市空间结构。围绕区域性中心城市定位，高水平抓好新一轮城市总体规划、城乡统筹规划、徐州都市圈规划修编和国土空间规划编制，加快推动“多规合一”，促进城市功能片区合理布局。注重控制强度、留出空间，科学划定开发边界和水体保护线、绿地系统线、基础设施建设控制线、历史文化保护线、永久基本农田红线、生态保护红线，适度拓展主城区面积和人口规模，推动城市发展由外延扩张式向内涵提升式转变。注重整体有序、联动开发，同步抓好老城区改造提升、新城区设施配套和开发区产城融合发展，支持鼓楼区老工业区搬迁改造、泉山区淮海生态新城建设、云龙区省级服务业综合改革试点、新城区商圈和金融集聚区建设，加快铜山、贾汪融入主城区步伐，形成组团发展的良好格局。注重传承历史、彰显特色，加强对城市的空间立体性、平面协调性、风貌整体性、文脉延续性等方面的规划和管控，留住徐州特有的地域环境、文化特色、建筑风格等“基因”。

完善提升城市功能。立足增强区域性中心城市吸引力和集聚力，以190项城建重点工程为抓手，提升城市的综合服务功能、设施配套水平和辐射带动作用。加快实施郑徐客专、徐连客专、徐宿淮盐铁路、观音机场二期等重大交通设施项目，加强徐州与周边城市的互联互通，巩固综合交通枢纽地位。继续抓好中心商圈扩容提质，积极发展高端业态，打造辐射周边的商贸中心。深度整合现有物流资源，大力推进现代物流基地建设，发展铁、水、公、空多式联运，健全现代综合物流体系。完善金融设施和布局规划，抓好金融集聚区和服务体系建设，构建区域性金融服务高地。突出抓好特色历史文化街区建设，精心打造重点文化园区和旅游景区，进一步丰富汉文化旅游节、文博会等节会内容，积极发展休闲养生、生态旅游，创建文化强市和旅游强市。继续抓好区域性教育、医疗中心建设，积极发展养老、健康等生活性服务业，满足群众日益增长的公共服务需求。探索土地成片整理、收储、利用方式，提高土地市场运作效益，促进房地产业健康发展。实施五山公园、泉润公园等城市园林景观工程，开展三环北路、新元大道等沿路沿河绿化，构建绿色生态网络体系，让群众共享“绿色福利”。

提升城市管理水平。以创建全国文明城市为总抓手，建立与现代化城市相适应的城管体制机制，精心塑造美丽城市、精品城市。抓好重大基础设施建设，大力实施城市轨道交通、高架快速路和重点道路贯通工程，构建立体化、便捷化的交通体系，加快打通断头路、改善微循环，努力缓解城市交通拥堵；推进海绵城市和综合管廊建设试点，启动第二垃圾焚烧发电厂、餐厨废弃物处理场等环卫设施建设，为城市管理提供良好硬件支撑。大力开展市容市貌综合整治行动，深入实施占道经营、交通秩序、违章建筑、物业管理、老旧小区等专项整治，进一步加大机电动三（四）轮车非法营运整治力度，切实有效治理“脏、乱、差”现象。推动互联网、大数据与城市管理深度融合，构建地理信息、智能交通、市容环境管理、灾害应急处置等智能数字化系统，抓好跨地区、跨部门、跨行业信息资源整合共享，加快打造“智慧徐州”。围绕构建长效管理机制，积极稳妥推进城市综合执法体制改革，推广“城管+公安”巡查一体、执法协作模式，完善环卫保洁、道路养护等市场化运作机制，持续开展示范路、示范社区和幸福家园创建活动，不断提升城市管理长效化、科学化、精细化水平。

（三）以推进城乡一体化发展为目标，不断加快新型城镇化进程，切实推动城乡统筹协调发展。坚持以工业化带动城镇化，促进县域二三产业充分发展，抓好镇村规划建设，推动农村劳动力加快转移，不断缩小城乡差距、提升城镇化质量和水平。

进一步做强区域经济。加强“一县一策”分类指导，落实好新一轮“丰县崛起”、“突破睢宁”、“沛北一体化”等政策意见，加快邳州新型工业化进程和新沂国家中小城市综合改革试点进度。积极借鉴先进发达地区在产业招商和产业链培育上的有效做法，引导各县（市）区因地制宜抓好招商引资工作，确保各地每年新上一批10亿元以上的产业项目，加快培育具有良好市场空间和发展潜力的特色产业集群。完善县（市）区科学发展综合考核体系，推动各县（市）区始终保持争先进位、竞相发展的浓厚氛围。

构建更加合理的城镇体系。抓好各县（市）总投资178亿元的40项城建重点工程，加强5个县级中等城市建设，增强产业发展、人口集聚、吸纳就业和公共服务等功能，加快打造宜居宜业的中等城市。按照特色化、差异化的思路，继续做好重点中心镇、特色小城镇建设和管理示范镇创建，完成12个重点中心镇环境整治任务，年内创建5个以上的达标重点中心镇和特色小城镇，考核命名第三批管理示范镇。建设镇村公路200公里，提升城乡客运一体化水平，统筹抓好区域供水、垃圾处置、污水处理等基础设施建设，推动基本公共服务向镇村延伸。

大力推进美丽乡村建设。遵循村镇人口变动趋势，根据各村区位、规模、发展条件、风貌特色等情况，合理确定村庄布点和建设规模，稳妥推进村庄撤并，加快形成适度集聚、生产便捷、生活舒适的村庄分布格局。深入推进村庄环境优化提升行动，完善农村卫生保洁和垃圾收运长效管理机制，抓好农村危房改造和排污设施建设，硬化350万平方米村组道路，建成一批新农村示范村，创建一批省级“康居村庄”和“美丽村庄”，进一步改善农村居住条件。年度投资10.3亿元，加大农田水利和农村水环境建设力度，解决好农村127万人饮水安全问题。

（四）以强化发展动力为目标，不断深化改革开放创新，为经济发展提供新的支撑。改革、开放、创新是新常态下区域发展的新动力。要紧紧围绕供给侧结构性改革方向，以深化改革优化体制机制，以扩大开放集聚生产要素，以创新驱动提升发展水平，持续激发经济发展活力。

坚定不移推进改革攻坚。落实具有重大牵引作用的改革举措，以深化行政管理体制改革为引领，带动其他领域改革。深化财税金融改革，建立全面规范、公开透明的预算制度，抓好综合治税，整合盘活各类资金，做大产业基金规模，培育壮大市级投融资服务平台，推进政府存量债务置换。深化国有企业改革，完善国有资产管理体制，促进国有资本向新兴产业和主导产业中高端集中，积极推进国有企业、国有资本采取混合所有制方式开展增量改革重组。深化商事制度改革，巩固和扩大“三证合一”、“一照一码”改革成果，加快工商注册便利化。深化农村改革，完成农村土地承包经营权确权登记颁证，抓好农村产权交易市场试点，稳妥推进农村集体经营性建设用地、农村宅基地、征地制度改革，加快推进不动产统一登记步伐，逐步将林地、农村土地承包经营权等纳入不动产统一登记范畴。深化户籍制度改革，降低城镇落户门槛，促进有能力在城镇稳定就业和生活的常住人口有序实现市民化。

深入推进创新驱动发展。积极推进大众创业万众创新，完善扶持政策，抓好众创空间建设，举办创新创业大赛，选树创新创业典型，开展成果展示、项目对接、创业辅导等主题活动。发挥企业创新主体作用，支持企业建设各类研发机构、组建创新联盟、开展技术创新和科技成果转化。提升矿大科技园等国家级创新平台的孵化能力，完善中关村信息谷创新中心、好欣晴移动医疗平台、鼓楼创客邦孵化器、邳州非晶科技企业孵化园等新载体功能，大力培育科技型中小微企业。坚持招才引智和自主培养相结合，继续实施“彭城英才计划”，培育引进一批高层次人才和创新创业团队，新增一批博士后、院士工作站，引进诺贝尔奖得主在我市设立研究院。财政要强化政策激励和扶持，加大对基础性科研的投入，引导社会特别是企业加大对科技创新的投入，不断提高科研经费占GDP的比重。创新融资方式，推广运用PPP模式，鼓励发行养老产业、创新创投和停车场等专项企业债，扩大直接融资总量和比重。

全面提升对外开放水平。注重对内对外开放并举、引资和引智并重、引进来和走出去联动，加快高水平双向开放步伐。把招商引资放到更加突出位置，主攻项目招商，坚持领导招商，加强招商队伍建设，完善招商激励措施，开展务实高效的点对点、小分队、巡回式招商，注重产业链招商和以商引商，办好一系列重点招商活动，努力在全市掀起招商引资的新热潮，实现引资和重大项目建设的新突破。做好工程机械、光伏、板材等重点出口行业的动态跟踪和预警分析，抓好保税物流中心管理运营，推进铜山货场一类口岸建设，继续打造一批出口基地和出口品牌，稳定对外贸易规模，优化出口产品结构，发展跨境电商业务。主动对接、参与、融入“一带一路”国家战略，支持徐工、徐矿等优势企业开展境外投资，扶持有条件的企业“走出去”。创新开发区体制机制和运营模式，推进徐州经济技术开发区“三次创业”，完善高新区功能配套，引导各省级开发区错位发展，支持沛县、邳州、新沂开发区创建国家级开发区，鼓励睢宁临空产业园、利国循环经济产业园争创省级开发园区，打造经济发展的主阵地、对外开放的高端平台。

（五）以增进民生福祉为目标，不断提升居民生活水平，增强人民群众获得感。始终把改善民生作为政府工作的重要任务，精心实施72项为民办实事项目，稳步提高城乡居民收入、社会保障水平、公共服务能力，让民生改善成果更多惠及人民群众。

积极拓宽居民增收渠道。健全工资水平决定机制、正常增长机制、支付保障机制，完善企业工资指导线和最低工资制度，合理调整最低工资标准，重点解决农民工工资支付保障问题，努力提高居民工资性收入。深入实施全民创业工程，构建创业指导帮扶和服务保障体系，抓好大学毕业生、复转军人等重点群体创业，不断增加居民经营性收入。深入开展用工对接，抓好技能培训和就业培训，积极开发就业岗位，大力解决就业结构性矛盾，力争年内城镇新增就业8万人、城镇失业人员再就业3.6万人、农村劳动力转移4万人，多层次多渠道扩大就业收入。

切实做好新一轮扶贫开发。按照6000元的扶贫新标准，摸清底数，建档立卡，加快推进69万低收入人口增收脱贫，年内确保完成17.5万人的脱贫攻坚任务。认真贯彻标准再提高、重点再聚焦、内涵再丰富、底线再织牢的工作要求，坚持精准扶贫、精准脱贫，因人因户分类施策，推进低收入农户结对帮扶全覆盖，有效提升脱贫的精准度和实效性。抓好黄河故道沿线、湖西革命老区2个扶贫开发重点片区的综合开发、整体帮扶，集中力量发展长效稳定的帮扶项目。把发展村级集体经济摆在突出位置，因地制宜推进216个经济薄弱村加快发展，尽早实现新“八有”目标。充分发动机关、社会组织、企业对口挂钩帮扶薄弱村和贫困户，引导党员结对帮助困难群众，通过帮扶不仅使贫困群众脱贫，更要在全社会形成互相帮助、助人为乐的良好社会风尚。

继续提升社会保障和救助水平。大力推进全民参保登记计划，全面实施机关事业单位养老保险改革，发行60万张加载金融功能的社保卡。加快建立城乡统一的基本医疗保险制度，健全医疗保险筹资和报销比例调整机制，实现基本医疗保险、大病保险与医疗救助有机衔接。实施860万平方米棚户区（危旧房）、城中村改造，统筹推进公租房和廉租房并轨运行、住房保障动态标准调整、经济适用房上市交易等工作，扩大住房公积金制度覆盖面，加快建立购租并举的住房制度。逐步为全市老人、残疾人和残疾儿童办理意外伤害保险。提高“三无”老人、孤儿供养以及县域尊老金发放标准，结对帮扶1.3万名困境儿童，为5000名经济困难或患有重大疾病的儿童提供经济帮扶。

推动各项社会事业均衡发展。深化教育改革，增强教育活力，提高教育质量。新改扩建110所幼儿园，创建省市优质园55所。新建续建35所中小学校，重建和加固36.6万平方米校舍，创建100所义务教育现代化学校。推进中高职衔接试点，创建一批省现代化示范性中等职业学校、实训基地和专业群。支持市内高校加快建设全国一流大学和一流学科。深化公立医院综合改革，探索分级诊疗，鼓励社会办医。加快推进第一医院迁建、市中医院改扩建和北区股份制医院建设等工程，提高乡镇卫生室、社区卫生服务中心医疗条件和服务能力，努力打造“健康徐州”。实施全面二孩政策，综合治理出生人口性别比失调问题。建设文化强市，大力推进文化事业发展，深入实施“舞动汉风”工程，抓好市区两级文化馆及市数字档案馆建设，全面推进新一轮公共文化设施达标建设，稳步提升公共文化服务标准化、均等化水平。促进文化繁荣，培育更多的文化名人、精品力作，丰富群众文化生活。加快发展文化产业，逐步提高文化产业比重。举办第21届市运会和第2届全民健身运动会，有序更新老旧室外健身器材，建设公共体育服务体系示范区。大力发展老龄事业，加快建设以居家养老为基础、社区为依托、机构为补充的多层次养老服务体系。加强军民融合式发展，确保全国双拥模范城创建八连冠。继续深化殡葬改革，抓好殡葬综合整治。统筹抓好其他各项社会事业。

下大力气提升生态环境。继续实施“五大行动计划”，强化污染治理和生态修复，积极发展循环经济，扎实推进美丽徐州建设。抓好扬尘污染整治、燃煤污染削减、机动车船舶污染控制以及秸秆禁烧和综合利用等工作，继续降低PM2.5浓度、减少雾霾天数、提升空气质量。实施重点湖泊、王窑河及城区黑臭河道整治工程，建设三八河污水处理厂三期、龙亭污水处理厂二期等项目，持续改善水环境。统筹抓好农业面源污染、养殖业污染、土壤污染及杨絮污染治理。完成绿化造林4万亩，构建城乡绿色屏障。落实最严格的耕地保护制度，建立工业用地退出机制，努力盘活存量建设用地，促进节地水平和产出效益双提升。用好全国新一轮工矿废弃地复垦利用试点政策，大力实施黄河故道流域土地综合整治，抓好九里独立工矿区改造、庞庄采煤沉陷区治理2个国家级项目，变生态包袱为生态亮点。

（六）以推进治理体系和治理能力现代化为目标，不断提升社会治理水平，努力开创社会和谐稳定新局面。大力加强法治徐州、平安徐州和文明徐州建设，着力营造优质高效的政务环境、公平正义的法治环境、健康向上的人文环境和安全稳定的社会环境。

深化行政管理体制改革。实行政府职责清单化管理，构建大部门综合管理模式，深入实施事业单位分类改革，继续做好省级经济发达镇行政管理体制改革试点、市级扩权强镇试点以及街道办事处管理体制改革。以提升政府服务经济、治理社会效能为导向，坚持放管结合，深化行政审批制度改革，进一步削减审批事项，完善政府职权清单管理动态调整机制、部门审批协同机制和事中事后综合监管体系，不断释放市场活力和社会创造力。落实行业协会去行政化措施，集中开展行政审批中介服务专项治理。

全面推进依法治市。注重发挥法治的引领、导向和规范作用，突出依法行政、公正司法和全民守法，在全社会营造崇尚法治的浓厚氛围。认真贯彻《法治政府建设实施纲要（2015－2020年）》，加快构建职能科学、权责法定、执法严明、公开公正、廉洁高效、守法诚信的法治政府。健全依法决策机制，落实重大行政决策法定程序，提高专家论证和风险评估质量，确保决策制度科学、程序正当、过程公开、责任明确。稳妥开展综合执法改革试点，加强执法标准化建设，落实行政执法责任制和执法责任追究制，规范执法自由裁量权行使。自觉接受人大及其常委会的法律监督、工作监督和政协的民主监督，重视社会公众监督和舆论监督，加强层级监督、行政监察和审计监督，确保各项工作在法律框架内开展。推动市县司法机关创新工作模式，抓好公共法律服务体系建设，继续打造12348法律服务平台特色品牌。深化法治宣传教育，创新“七五”普法方式方法，增强全社会学法守法用法意识。

加强社会治理创新。抓好公民道德建设工程和群众性精神文明创建活动，大力培育社会主义核心价值观，深化“好人文化”建设，提高全民文明素质和科学素质，推动社会风气持续好转。抓好县镇村三级综合服务管理平台规范化建设，深入推进政社互动、“三社联动”和社区减负增效，加强社区干部队伍建设，提升人员素质，提高保障水平，改善工作条件，切实健全基层社会治理体系。加强社会治安综合治理，健全以信息化为支撑的社会治安防控体系，严厉打击各类刑事犯罪。完善徐州特色的多调对接、多元化矛盾纠纷调处机制，深化信访工作制度改革，依法依规协调和处理群众诉求。强化安全生产责任和管理制度落实，强化食品药品安全监管，完善产品质量检验检测平台和电梯应急救援平台，有效防范和坚决遏制重特大安全事故发生。加强应急体系建设，提高公共安全和防灾减灾能力。

（七）以构建高效廉洁的服务型政府为目标，不断加强政府自身建设，大力提升政府公信力凝聚力执行力。面对新形势、新任务，要把政府自身建设摆在突出位置，忠诚于党、忠诚于人民、忠诚于国家，把法治政府建设与效能型政府、服务型政府、廉洁型政府建设相结合，切实增强政府组织经济工作、加强社会管理、处置复杂问题、推动工作落实的能力。

高水平构建效能型政府。围绕提高施政能力，切实转变作风，引导政府系统干部树立正确的权力观、事业观、政绩观，出实招、办实事，更加理性、更加高效地推动各项工作落实。围绕提高执行能力，倡导“马上就办、真抓实干”，健全奖惩机制，加强绩效管理、政务督查和行政问责，充分调动各方面干事创业积极性，对上级重大决策部署和市委市政府确定的目标任务一抓到底、务求实效。围绕提高办事效率，加快电子政务建设，推进相对集中行政许可权改革试点，着力破解行政审批“一长四多”的问题。围绕规范行政权力运行，把政府工作全面纳入法治轨道，切实减少政府对微观经济活动的干预，为各类市场主体创造机会更加均等、竞争更加公平的市场环境。

高水平构建服务型政府。从践行群众路线入手，强化宗旨意识、为民意识和服务意识，深入基层察实情、办实事、求实效，坚持问政于民、问需于民、问计于民，切实解决群众反映强烈的突出问题。从优化政务服务入手，推进百姓办事“零障碍”工程向基层延伸，加快政务服务、公共资源交易等新中心整合建设，打造集行政许可服务、公共资源交易、政府服务热线等一体化管理运行的政务服务综合性平台。从抓好政务公开入手，完善政府信息发布制度，推进行政审批网与行政权力网上公开透明运行系统深度融合，深化“两参一改”群众评议，提高政府工作的透明度和公众参与度。从推进“诚信徐州”建设入手，加强政府诚信体系建设和企业信用等级分类管理，构建守信激励和失信惩戒机制，努力营造诚实守信的良好环境。

高水平构建廉洁型政府。严格遵守政治纪律、政治规矩，不断增强政治定力，自觉与党中央保持高度一致。自觉践行“三严三实”，坚持不懈纠正“四风”，从严从细落实中央“八项规定”和省委市委“十项规定”以及作风建设一系列部署要求，不断加强廉政教育，严肃整治“为官不为”、乱作为等问题，以作风转变的新成效取信于民。注重标本兼治，坚持用制度管权、管事、管人，紧紧扎住制度的“笼子”，促进行政权力规范公开透明运行。厉行勤俭节约，严控“三公”经费支出，严把财政资金支出审批关。启动事业单位、国有企业公车改革。严格落实党风廉政建设责任制，深入推进政府系统预防和惩治腐败体系建设，严肃查处各类违法违纪案件，保持严惩腐败的高压态势，不断增强人民群众对党和政府的信心、信任和信赖。

各位代表！徐州全面振兴的光荣使命鼓舞我们砥砺奋进，全市人民的信任期盼鞭策我们勇往直前。让我们紧密团结在以习近平同志为总书记的党中央周围，高举中国特色社会主义伟大旗帜，在中共徐州市委的坚强领导下，团结依靠全市人民，敢于担当，真抓实干，为“迈上新台阶、建设新徐州”而努力奋斗！
